# Infection in patchy populations: Contrasting pathogen invasion success and dispersal at varying times since host colonization

**DOI:** 10.1002/evl3.141

**Published:** 2019-09-24

**Authors:** Louise S. Nørgaard, Ben L. Phillips, Matthew D. Hall

**Affiliations:** ^1^ School of Biological Sciences Monash University Clayton Melbourne 3800 Australia; ^2^ School of BioSciences University of Melbourne Parkville Victoria 3010 Australia

**Keywords:** competitor versus colonizer, diversifying selection, dose–density response, *Daphnia magna*, evolution of disease, epidemiology, *Pasteuria ramosa*, spatial spread

## Abstract

Repeated extinction and recolonization events generate a landscape of host populations that vary in their time since colonization. Within this dynamic landscape, pathogens that excel at invading recently colonized host populations are not necessarily those that perform best in host populations at or near their carrying capacity, potentially giving rise to divergent selection for pathogen traits that mediate the invasion process. Rarely, however, has this contention been empirically tested. Using *Daphnia magna*, we explored how differences in the colonization history of a host population influence the invasion success of different genotypes of the pathogen *Pasteuria ramosa*. By partitioning the pathogen invasion process into a series of individual steps, we show that each pathogen optimizes invasion differently when encountering host populations that vary in their time since colonization. All pathogen genotypes were more likely to establish successfully in recently colonized host populations, but the production of transmission spores was typically maximized in either the subsequent growth or stationary phase of host colonization. Integrating across the first three pathogen invasion steps (initial establishment, proliferation, and secondary infection) revealed that overall pathogen invasion success (and its variance) was, nonetheless, highest in recently colonized host populations. However, only pathogens that were slow to kill their host were able to maximize host‐facilitated dispersal. This suggests that only a subset of pathogen genotypes—the less virulent and more dispersive—are more likely to encounter newly colonized host populations at the front of a range expansion or in metapopulations with high extinction rates. Our results suggest a fundamental trade‐off for a pathogen between dispersal and virulence, and evidence for higher invasion success in younger host populations, a finding with clear implications for pathogen evolution in spatiotemporally dynamic settings.

**Impact summary**

Infectious disease is a major threat to human and wildlife well‐being, and it is well recognized that pathogens have the ability to evolve over time to persist in a host population. Host–pathogen theory has largely centered on the evolution of disease in stable and nondynamic host populations. However, natural host populations are rarely static and rather characterized by changing population dynamics (and densities) across time and space. For example, in range expanding species, or analogous metapopulations with high extinction rates, empty habitat is regularly colonized by dispersing individuals. Thus, understanding how infectious disease evolves as it spreads through a patchy landscape, and what pathogen strategies may be favored at different times of the host colonization process, is essential for understanding the evolution of pathogens in nature.

Using the waterflea *Daphnia magna* and its associated bacterial pathogen *Pasteuria ramosa*, we experimentally tested the idea that different pathogen strategies may be favored whenever pathogens encounter host populations that vary in their time since colonization. Our results show that pathogen genotypes optimized the invasion process in different ways, and an invasion strategy that is successful when invading newly colonized host populations is unlikely to be successful when encountering host populations undergoing the subsequent growth or stationary phases. In particular, the ability of a pathogen to disperse via its infected carrier determines whether a pathogen is likely to coincide with newly colonized host populations. Our results suggest that prudent and highly dispersive pathogens are more likely to encounter (or keep pace with) newly colonized host populations at the range front of an expanding host population or in metapopulations. Our work demonstrates that pathogen invasion success and spatial spread is strongly linked to the host population dynamics encountered. This empirical work thus reiterates the importance of developing a better understanding of how host population dynamics influences the evolution of infectious disease in spatially explicit settings.

In 1951, GE Hutchinson coined the term “fugitive species” for species that are specialized colonizers (Hutchinson 1951). These species, he argued, while good at colonizing empty patches, are typically poor competitors. As such, they exist in a tenuous niche, constantly needing to colonies newly available space before they are outcompeted in their current location by later arriving, but competitively superior species (Calcagno et al. 2006). Although originally applied to the distribution of free‐living species across a patchy landscape (Tilman 1994), tension between colonization and competition is emerging as a key modifier of pathogen life history as well. Studies have observed, for example, that the initial performance of a pathogen after invading a host population does not necessarily translate into long‐term persistence (Fellous et al. 2012), that migratory animals can have a lower pathogen prevalence than their nonmigratory relatives (Bradley and Altizer 2005), and that pathogens can lag behind their hosts during periods of host range expansion (Boots et al. 2004; Phillips et al. 2010; Fellous et al. 2011). Collectively, these examples imply a fundamental trade‐off between pathogen performance in established host populations versus their capacity to disperse to, and establish in, new patches, such that an invading pathogen genotype will rarely, if ever, be good at both.

Conditions that favor different pathogen establishment or competitor strategies are routinely met in host metapopulations (Ebenhard 1991; Hanski and Gilpin 1991; Soubeyrand and Laine 2017). Here, stochastic extinction at the patch level generates vacant habitat available for recolonization by dispersing hosts, resulting in a landscape of populations at different colonization phases. Typically, populations undergo an exponential growth phase just after colonization, through to density‐regulated dynamics in older populations at or near the carrying capacity, possibly with some demographic overshoot in between (Gilpin and Ayala 1973; Hanski and Gilpin 1991; Drake and Griffen 2009). A similar range of demographic scenarios exist in a host population spreading through space, where the expansion front is growing exponentially (Giometto et al. 2014), and the core parts of the population are often density regulated (Sakai et al. 2001; Dwyer and Morris 2006; Phillips, 2009, 2015; Sullivan et al. 2017).

In general, opportunities for diversifying selection to act on a pathogen will occur organically as a consequence of the invasion process itself. Invasion, much like other complex processes such as infection (Hall et al. 2017) or zoonotic spillover (Plowright et al. 2017), requires a pathogen to overcome multiple barriers to successfully reproduce, transmit, and disperse (see Fig. 1; sensu Blackburn et al. 2011). For an outbreak of infectious disease to occur, a pathogen must first establish in the host population after an infected carrier or propagule arrives (stage 1). It must then proliferate inside the infected host (stage 2) and spread the infection to new susceptible hosts (secondary infection) within the same population (stage 3). Finally, the pathogen must also disperse to new patches (passively or, for many pathogens, via host movement) to infect new host populations in a patchy landscape, or keep pace with the rate of its host's range expansion (stage 4). Viewing this process through the lens of diversifying selection implies that the demographic conditions in any given host patch will define what is optimal for a given pathogen genotype at each stage of the invasion process, and in turn, the types of pathogen strategies that are likely to be favored in any given patch or time (e.g., Wei and Krone 2005).

**Figure 1:**
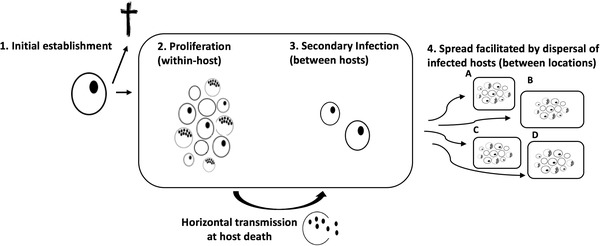
The four steps that a pathogen faces to successfully establish and spread in host populations. (1) A pathogen must successfully establish in a new susceptible host (circle) after an infected carrier or propagule enters a host population; (2) proliferation and production of mature transmission spores (black filled dots) within infected host; (3) generate secondary infections (after host death) in new susceptible hosts within the same population; and finally, (4) spread between populations to infect new host populations in a patchy landscape, or keep pace with the rate of its range expanding hosts. Host growth trajectories and population density influences each pathogen invasion step, and will define what is optimal for a pathogen at each stage of its invasion process.

Theory describing the types of pathogen strategies that are favored during the invasion process has traditionally focused on long‐term changes in selection. Highly transmissible and virulent pathogens, for example, are expected to prevail at the start of the invasion (i.e., epidemic phase), whereas more prudent exploitation strategies are favored once a pathogen is maintained stably in the host population (i.e., endemic phase) (e.g., Lenski and May 1994; Day and Proulx 2004; Day and Gandon 2007; Berngruber et al. 2013). These predictions, however, center only on one part of a pathogen's invasion process (i.e., stages 2 and 3, Fig. 1) and the long‐term evolutionary dynamics of pathogens in isolated and demographically stable host populations. Instead, trade‐offs between the damage a pathogen causes its host versus its capacity to disperse into new patches (steps 2 and 3 vs. 4, Fig. 1) have emerged as the defining characteristic of how a pathogen might evolve when host populations vary in time and space (Griette et al. 2015; Osnas et al. 2015). When trade‐offs between dispersal and virulence occur, theory predicts that evolution will favor more prudent host exploitation strategies at the expanding range edge (Osnas et al. 2015). In the absence of such a trade‐off, highly virulent pathogen strategies are expected to prevail at the range edge (Griette et al. 2015).

Accurate predictions for the rate at which a pathogen spreads through space, or how it might evolve during an invasion, thus depend on both the demography of a host patch and the occurrence of any trade‐offs between exploitation and dispersal. Yet, few studies have directly estimated whether different pathogen genotypes diverge along such a colonizer‐competitor continuum. Here, we used the waterflea *Daphnia magna* and its bacterial pathogen, *Pasteuria ramosa* (Ebert et al. 2016), to explore the sensitivity of each step of a pathogen's invasion (i.e., Fig. 1) to changes in the demography of the host population. *Daphnia* are an excellent candidate for exploring the effects of population dynamics, as habitat patches are commonly recolonized in response to seasonal changes, leading to large fluctuations in population density and disease prevalence over time (e.g., Bieger and Ebert 2009; Altermatt and Ebert 2010). The fitness of *P. ramosa* is also tied to the demography of its host as transmission depends on both virulence (i.e., host mortality) and the production of transmission spores (see Hall and Mideo 2018), both of which respond to changes in host density (Ebert et al. 2000; Michel et al. 2016). However, little is known about how infection alters host movement and subsequent spatial spread of disease.

With this system, we experimentally invaded multiple pathogen genotypes into host populations at different colonization phases, and evaluated whether pathogen genotypes varied along the competition‐colonization axis that is integral to recent models of pathogen invasion (Griette et al. 2015; Osnas et al. 2015). First, we demonstrated that host populations possess three clear (and repeatable) population growth phases following initial colonization (rapid growth after initial colonization, overshoot, and stationary phase). Next, in a series of experiments, we showed how each step of a pathogen's invasion (i.e., Fig. 1; initial establishment, proliferation, secondary infection) is highly sensitive to the colonization phase of the host population experienced and differs between pathogen genotypes. Finally, using a simple epidemiological model, we integrated each component of a pathogen's invasion into a single metric of invasion success at each host colonization phase, and compared this with their ability to disperse. Our invasion metric and dispersal measures supported the prediction that diversifying selection may arise as a natural consequence of host population colonization. Here, pathogens will either be good at invading established host populations or good at dispersing via movement of infected carriers. With this in mind, we discuss how changes in host colonization dynamics can provide the conditions that should favor different colonization or competitor strategies in patchy landscapes, and the implications this has for the evolution of pathogen virulence.

## Materials and Methods

### THE STUDY SYSTEM

*Daphnia magna* Straus is a filter‐feeding cladoceran that reproduces via cyclic parthenogenesis (matures at approximately 10 days old, Ebert 2005). It is found in freshwater brackish lakes and ponds throughout Eurasia and North America and populations experiences large fluctuations in density throughout a year (Ebert 2005; Altermatt and Ebert 2010). A common pathogen of *Daphnia* is the endospore forming, Gram‐positive bacteria, *Pasteuria ramosa* Metchnikoff, 1888. *Daphnia* encounter *P. ramosa* through filter feeding, after which spores start to proliferate and the first mature transmission spores appears about 18 days post infection (DPI), at which point infection is easily recognized due to a change in the coloration of the infected host (Ebert et al. 2016). Transmission is exclusively horizontal, and symptoms of disease includes castration, gigantism, and reduced lifespan (Hall and Ebert 2012; Ebert et al. 2016). The host used in this study originated from Hungary (HU‐HO‐2), and the five novel *P. ramosa* genotypes originated from Russia (C1), Finland (C14), Germany (C19), UK (C20), and Belgium (C24), representing a wide geographical and ecological distribution (Luijckx et al. 2011). These five pathogen genotypes vary considerably in virulence (i.e., the reduction of host lifespan) and production of transmission spores (Clerc et al. 2015; Hall and Mideo 2018).

### OVERVIEW OF EXPERIMENTAL DESIGN AND CONDITIONS

This study consists of five sections: (1) first, we characterized the dynamics of host colonization within a multipatch system by tracking changes in population size over time within each patch; (2) we then measured how the first two steps of pathogen invasion for multiple pathogen genotypes (establishment and proliferation, Fig. 1), varied with host colonization phase; (3) we estimated how infection rates at different host densities relate to the number of transmission spores potentially released from an infected carrier; (4) we measured the impact of infection on host dispersal; and finally, (5) we integrated the above pathogen traits (except for dispersal) into a single metric of pathogen invasion success. Unless stated otherwise, all experiments were maintained following standard protocols and growing conditions as outlined in the Supporting Information (section A).

### SECTION 1: CHARACTERIZING HOST COLONIZATION DYNAMICS

**Figure 2:**
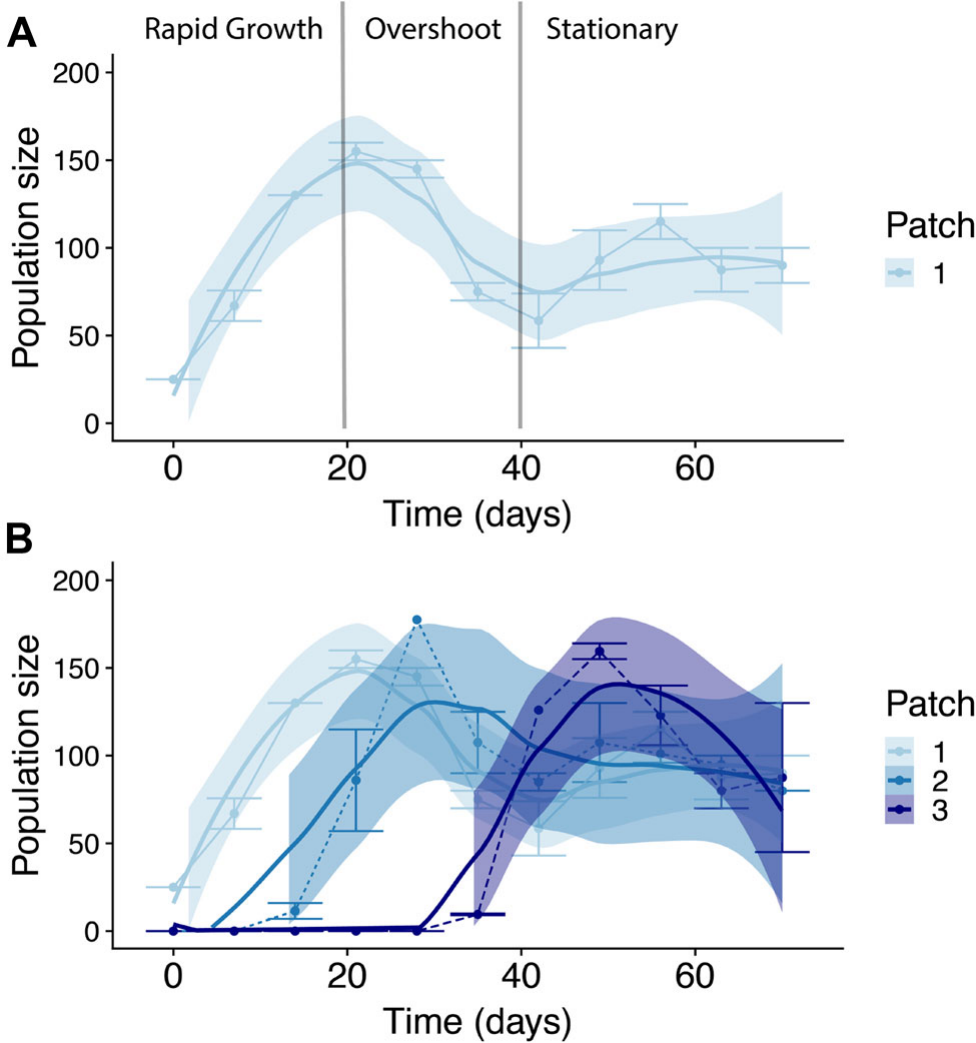
Repeated colonization events of *Daphnia* populations (female and juveniles) in interconnected experimental patches. (A) A population undergoing different colonization phases in a single habitat patch, defined as (i) a period of rapid population growth following initial establishment, (ii) a short period of population overshoot, and (iii) density‐regulated carrying capacity or stationary phase. In our interconnected habitats, dispersers move from an established patch to a new one, after a time‐lag of approximately 14 days, and the characteristic colonization phases are then repeated in time and space (B). Colored‐filled lines represent fitted GAM predictions of population size for each interconnected patch, and shaded area depicts 95% confidence intervals. Thin dotted lines represent raw data and corresponding error bars represent standard errors. Written annotations and lines in (A) visualize the population colonization phases (i–iii) for patch 1.

To determine the repeatability of host colonization, we first created four replicates of three interconnected experimental patches built from 500‐mL containers filled with 400 mL artificial media (see Erm et al. 2019). On day 1, five 3‐day old juveniles and one adult *Daphnia* were introduced into the first patch, and the subsequent population sizes at each patch were counted weekly thereafter. Different age‐classes were incorporated to recreate more natural populations with overlapping generations. Populations were fed 50 million green algae (*Scenedesmus* sp.) cells three times a week and 50% of the media was renewed fortnightly. After 70 days, when all patches were inhabited, the experiment was terminated. From these results, we defined three repeatable characteristics of the colonization process: rapid growth after initial colonization, overshooting the carrying capacity from day 20, and stationary phase from day 40 onwards (see Results section, Fig. 2, and Supporting Information, section B).

### SECTION 2: CHARACTERIZING PATHOGEN ESTABLISHMENT AND WITHIN‐HOST PROLIFERATION

We then tested how conditions in host populations undergoing these different phases of colonization (rapid growth, overshoot, and stationary), influenced the ability of a pathogen to successfully establish in a given host population and proliferate within their infected host (steps 1 and 2, Fig. 1). A total of 240 experimental populations were established using 500‐mL glass jars. Each population was fed three times a week and had 50% of their media renewed fortnightly. Populations were allowed to colonize for either 40, 20, or 0 days prior to introducing six 10‐day‐old *Daphnia*, infected with one of the five pathogen genotypes. Each pathogen genotype and host colonization phase were replicated 16 times ([3 host colonization phases × 5 pathogen genotypes × 16 replicates = 240 experimental populations] × 6 individuals introduced per population = 1440 infected individuals).

Deaths of the infected *Daphnia* introduced to experimental populations were monitored daily and cadavers were frozen individually in vials filled with 500 µL RO (removing the chance for secondary infections to arise by horizontal transmission). After 20 days (at which point all surviving *Daphnia* will have produced mature transmission spores, Clerc et al. 2015), all surviving *Daphnia* (infected and uninfected) were collected, checked for infection under a dissecting microscope, and frozen individually as described above. Spore loads of infected animals were later quantified using an Accuri C6 flow cytometer (BD Biosciences, San Jose, California) following standard procedures as outlined in Gipson and Hall, (2018). This procedure allowed us to estimate (1) the probability that the introduced infected *Daphnia* survive long enough for the pathogen to produce mature transmission spores within an infected host ($E_{dg}$, based on the proportion of recovered infected hosts that contained mature spores, where *dg* denote demographic phase and pathogen genotype); and, (2) pathogen transmission potential ($L_{dg}$, calculated as mean spore count within each host colonization phase and pathogen genotype).

### SECTION 3: CHARACTERIZING THE POTENTIAL FOR SECONDARY INFECTIONS

Following successful establishment and within‐host proliferation, the number of secondary infections immediately gained from the release of spores from a single cadaver depends on the number of infective spores released and the density of hosts in a given patch (stage 3, Fig. 1). We tested the influence of spore dose and host density on infection rates in a fully factorial setup using five environmentally relevant densities of *D. magna* (20, 80, 160, 240, or 320 individuals L^−1^; *n* = 20, 12, 8, 6, and 4) and three different doses of the five *P. ramosa* genotypes (50 000, 150 000, and 450 000 spores; i.e., low, medium, and high).

To establish the density treatments, the number of individuals required to create the densities (1, 4, 8, 12, and 16 individuals) were raised from birth in 50 mL ADaM. At 7 days old, the host density treatments were exposed to one of the three pathogen spore doses for 24 hours. After the exposure period, animals were transferred to jars with fresh media, at a standard density of four individuals per jar (within same density and pathogen combination) to maintain identical rearing conditions (and food availability) for all treatments. Experimental jars were then checked daily for deaths, and infection status was determined at 25 DPI based on the presence of mature spores. Due to mortality, we obtained infection status for 3857 out of 4020 individuals. Finally, assuming a density‐dependent transmission model, we use these data (proportion of individuals infected at each host density and pathogen spore dose) to estimate the transmission coefficient for each pathogen genotype for use in our index of pathogen invasion success ($\beta_{g}$; see Supporting Information).

### SECTION 4: EXPERIMENTAL MEASURES OF PATHOGEN DISPERSAL

To measure dispersal behavior of infected *Daphnia* and the consequent spread of *P. ramosa*, we introduced a single infected host (or uninfected control) into individual interconnected three‐patch systems, built from 50‐mL falcon tubes connected by 10 cm of food‐graded silicon tubing. Dispersal was only allowed in forward direction and when reaching the third patch, two additional patches (with fresh ADaM) were provided. Dispersal and survival were tracked daily, and at death, *Daphnia* were frozen individually in vials filled with 500 µL RO and stored at −20°C for later spore counts (as described above). Each infection treatment was replicated 20 times (5 pathogen genotypes + control × 20 replicates = 120 experimental replicates). We then estimated the rate at which individuals move through space (patches traveled per day), as well as the total dispersal distance (sum of patches traveled in a lifetime) for hosts infected with each pathogen genotype and uninfected controls.

### SECTION 5: AN INDEX OF PATHOGEN INVASION SUCCESS

Finally, we integrated each step of the pathogen invasion process (step 1, 2 and 3, Fig. 1) into an index of invasion success, allowing us to contrast the within‐patch exploitation versus between‐patch dispersal capacity of each pathogen genotype (step 4, Fig. 1). Our measure of invasion success for each pathogen genotype ($w_{dg}$, detailed below) captures the likelihood of generating new secondary infections within a population of susceptible hosts at a standard density (arbitrarily, *S* = 1). By standardizing host density, $w_{dg}$ focuses on relative fitness of pathogen genotypes, and diverges from the basic reproduction number of an infection (*R*_0_, Anderson and May 1981; Heffernan et al. 2005), which is often used to assess the rate at which a pathogen will invade a population. Our metric incorporates the uncertainty of a pathogen being able to establish in a patch after the initial introduction (step 1, Fig. 1). It also focuses purely on a pathogen's capacity to initiate an outbreak in a given population (i.e., the first secondary infection) rather than longer term persistence (which depends on host carrying capacity, birth rates, and death rates, as has previously been incorporated into estimates of *R*_0_ for infections in *Daphnia* by the fungal pathogen *Metschnikowia bicuspid*; Civitello et al. 2013; Dallas et al. 2018).

To estimate *w_dg_*, we first calculated the number of spores expected to be produced by each pathogen genotype in the three host colonization phases ($P_{dg}$, colonization phase *d* and genotype *g*), using data from Section 2. $P_{dg}$ results from multiplying the probability that an infected carrier establishes within a given patch (Edg, see  Section 2) by the subsequent mean number of mature transmission spores (Ldg, see  Section 2) that an infected individual would produce. We next used $P_{dg}$ to develop our measure of pathogen invasion success ($w_{dg}$) under the mass‐action assumption that the number of new infections would be $I_{dg}=\;\beta_{g}P_{dg}S$ (where *S* is an arbitrary host density and *β* is a transmission coefficient; see Supporting Information, section F). Here, $\beta_{g}$ is our independent estimate of transmission for each pathogen genotype, and is obtained from the data in Section 3, where $\beta_{gijk}=\frac{I_{gijk}}{P_{ik}S_{jk}}$ is the per‐replicate measure of *β*, where *I_gijk_* is the response variable (number of infected individuals), and *P_ik_* and *S_jk_* are the factor levels for pathogen dose and host density, respectively (*k* indexes the replicate within factor levels). We average this measure across *i, j, k*, to generate a genotype‐level estimate of *β_g_*, and use bootstrapping to capture uncertainty in this estimate. We then estimate the number of new infections at a standard density (*S* = 1) to provide a standardized metric of pathogen invasion success, ${\hat{w}}_{dg}=\;{\hat{\beta }}_{g}\;{\hat{P}}_{dg}$, for each combination of pathogen genotype and host colonization phase. We used the bootstrap distributions for *β* and *P* such that uncertainty propagated through to our estimate of *w* (see Supporting Information, section F).

### STATISTICAL ANALYSES

All statistical analyses were performed in R (version 3.4.1; R Development Team, available at www.r-project.org). In Section 1, we analyzed the host colonization data using a generalized additive model with days modeled as a thin plate spline (see Supporting Information). In Section 2, we used a linear and generalized linear mixed‐effect model, respectively, to analyze spore loads and the probability of establishment (using a binomial error structure). In each case, we used a two‐factor analysis of variance (Type III) with colonization phase (growth, overshoot, and stationary phase), pathogen genotype, and their interaction as fixed effects, and experimental jar as a random effect, as implemented via the lme4 package (Bates et al. 2015). In Section 3, we modeled the probability of infection using a generalized linear mixed‐effect model with a binomial error structure, as part of a two‐factor analysis of covariance (Type III) with pathogen genotype and spore dose (low, medium, and high) as fixed effects, host density as an interacting covariate, and experimental jar as a random effect. In Section 4, we analyzed dispersal behavior of infected hosts and uninfected controls in two different ways. First, as the total number of patches traveled (square root transformed before analysis) via an analysis of variance with infection treatment as a fixed effect. Second, we analyzed dispersal rate (accumulated patches traveled over time), as estimated via a linear mixed‐effects model with accumulated patches as response, infection treatment as fixed effect, time (in days) as an interacting covariate, and individual identity as random effect.

## Results

Experimental *Daphnia* populations undergoing colonization in patchy habitats displayed characteristic colonization trajectories that were repeatable in space (Fig. 2). Three broad transitions were evident: (1) a period of rapid population growth following the initial colonization; (2) an overshoot phase whereby a maximum population density is reached (with a brief decline as carrying capacity has been exceed); and finally, (3) a stationary phase where relatively stable population sizes at or around the carrying capacity is maintained (around 350 individuals liter^−1^, reflecting naturally observable population sizes; Kvam and Kleiven 1995). Importantly, colonization in the second and third patches followed the same demographic transitions, but with a time lag of ∼14 days. The demonstrated repeatability of colonization dynamics then allowed us to test how differences in colonization history may alter pathogen invasion success (Fig. 2 and Supporting Information, section B).

### PATHOGEN ESTABLISHMENT AND WITHIN‐HOST PROLIFERATION UNDER DIFFERENT HOST COLONIZATION PHASES

**Figure 3:**
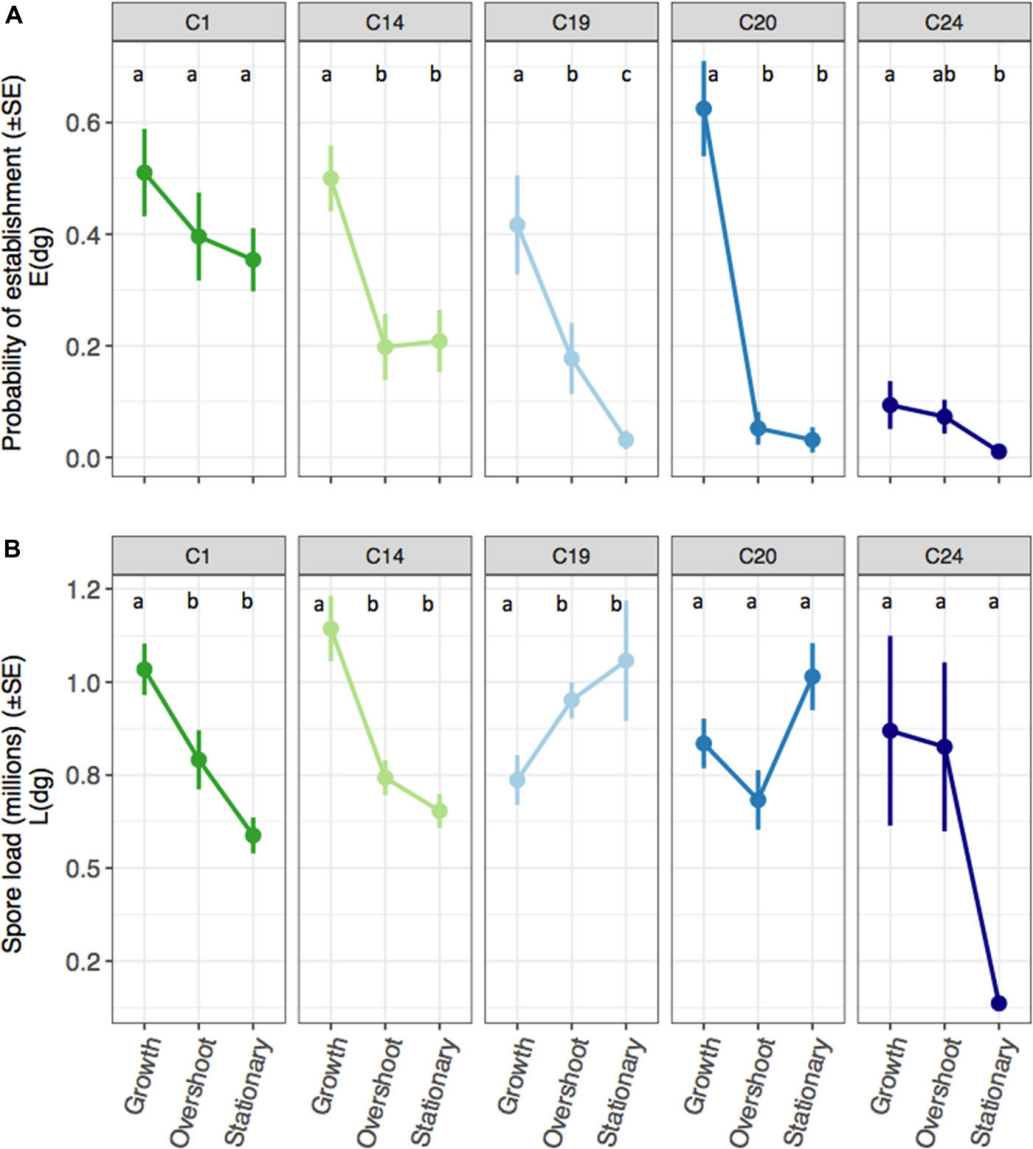
The influence of host colonization phase (rapid growth, overshoot, and stationary) on pathogen performance during the first two stages of the invasion process. (A) The probability that a pathogen successfully establishes and produces mature transmission spores (SE). (B) Pathogen spore load (transmission potential) in millions (SE) at host death or experimental termination. Letters refer to Tukey's post hoc significance test between host colonization phases within each pathogen genotype.

The ability of a pathogen to establish in a new patch (by surviving long enough to produce mature spores) and its subsequent transmission potential (spore production in hosts that successfully established) was highest when confronted with host populations undergoing rapid growth (Fig. 3). The exact responses, however, depended on a pathogen's genotype as evidenced by a significant genotype by host colonization phase interaction for both traits (probability of establishment: d.f. = 8, *χ* ^2^ = 31.853, *P* < 0.001; spore load in established infections: d.f. = 8, *χ* ^2^ = 20.310, *P* < 0.001; see Supporting Information, section C). Pathogen C1 and C14, for example, maximized both traits when introduced to populations undergoing rapid growth, but less so as the population exceed or reached carrying capacity. In contrast, the responses of pathogen C19 and C20 were trait specific; the probability of establishment was highest in host populations undergoing rapid growth, but production of transmission spores peaked instead in host populations at stationary phase.

### OPPORTUNITIES FOR SECONDARY INFECTIONS AND SUBSEQUENT DISPERSAL INTO NEW PATCHES

**Figure 4:**
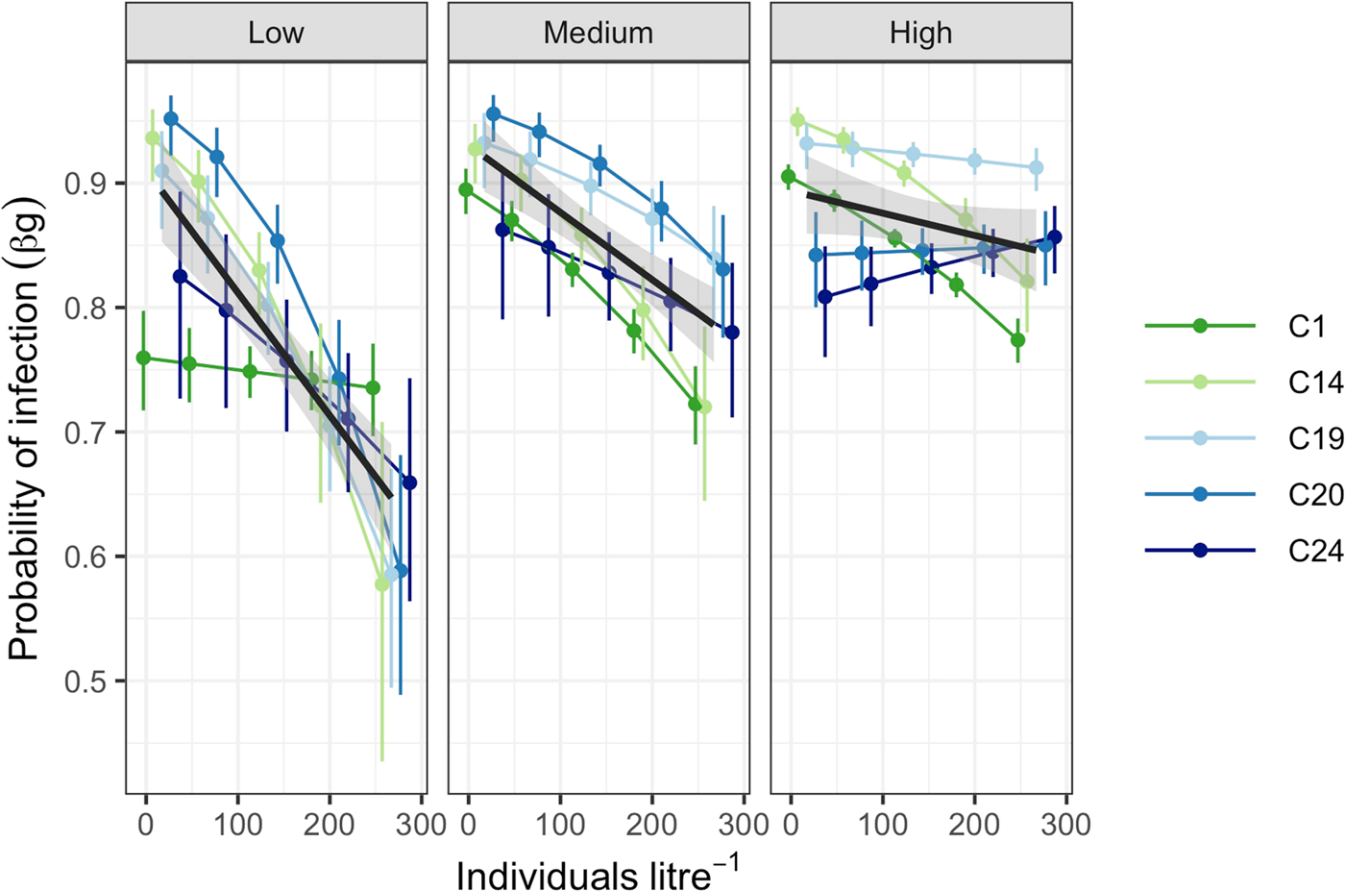
The influence of host density on the probability of infection (as per step 3, Fig. 1). Shown is the probability of infection at different population densities, exposed to three doses (low, medium, and high) of *P. ramosa* spores. In color are the predicted values with associated confidence intervals based on a fitted generalized mixed‐effect model, for each pathogen genotype, and the black line with gray shading depicts the overall trends (95% CI) across all pathogen genotypes.

We next conducted an experiment to estimate the ability of a pathogen to secondarily infect susceptible hosts at different densities (steps 3, Fig. 1). We found a decreasing probability of infection with increasing host density (Fig. 4), but this trend was modified both by pathogen genotype (d.f. = 4, *χ* ^2^ = 15.959, *P* < 0.003) and a dose‐by‐density interaction (d.f. = 2, *χ* ^2^ = 6.522, *P* = 0.038). This decline in infection rate was strongest at low spore dose; increasing the number of hosts in a fixed volume presumably dilutes the inoculation that each host effectively receives, to a point where infection is no longer completely assured (infection rates are known to increase in a dose‐dependent manner; sensu Ben‐Ami et al. 2008). Thus, at higher spore doses, this dilution effect is reduced.

**Figure 5:**
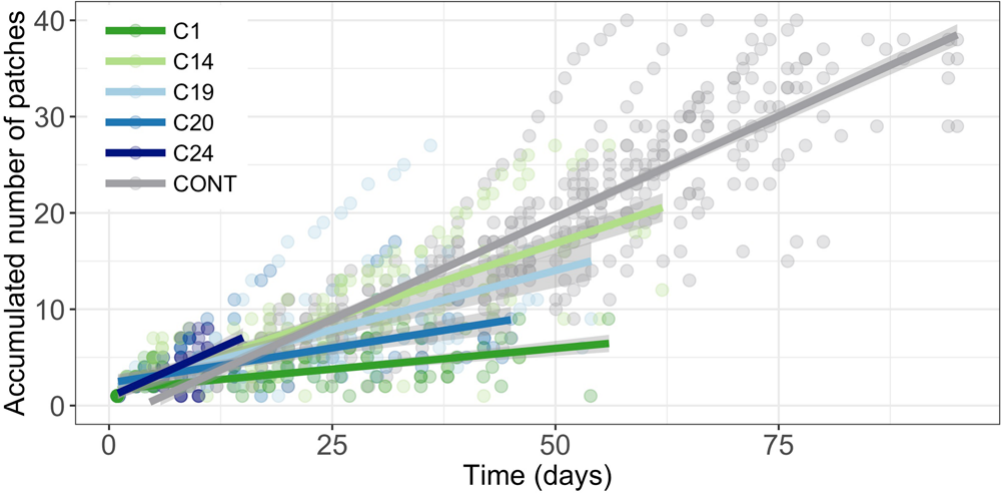
Host‐facilitated dispersal of pathogen genotypes and uninfected hosts. Shown is the accumulated number of patches traveled over time (days) in interconnected patchy habitats. Differences in length of the curves indicates the average lifespan of infected animals (pathogen induced reduction in lifespan) and uninfected controls. Dots represent individual replicate dispersal. Gray‐shaded area represents 95% confidence interval associated with model predictions.

Finally, a pathogen must disperse to invade new susceptible host populations (step 4, Fig. 1). Here, infection reduced the dispersal capacity of a host (Fig. 5), with significant variability both in the total distance covered among parasite genotypes and their uninfected counterparts (d.f. = 5, 111, *F*‐value = 35.662, *P* < 0.001), and in the rate at which animals moved through space (slope by infection treatment interaction: d.f. = 5, *χ* ^2^ = 445.114, *P* < 0.001). Across all infection treatments, the total distance covered (*r* = 0.816, d.f. = 112, 95% CI = 0.743–0.869, *P* < 0.001) and dispersal rate (*r* = 0.304, d.f. = 112, 95% CI = 0.127–0.462, *P* < 0.001) was positively correlated with the lifespan of an individual, albeit only weakly in the case of dispersal rate. More virulent pathogens (i.e., shorter host lifespan; Gipson and Hall 2018), therefore, appear to curtail the movement of the host on which their dispersal depends.

### CONTRASTING INTEGRATIVE PATHOGEN INVASION SUCCESS WITH DISPERSAL CAPACITY

**Figure 6:**
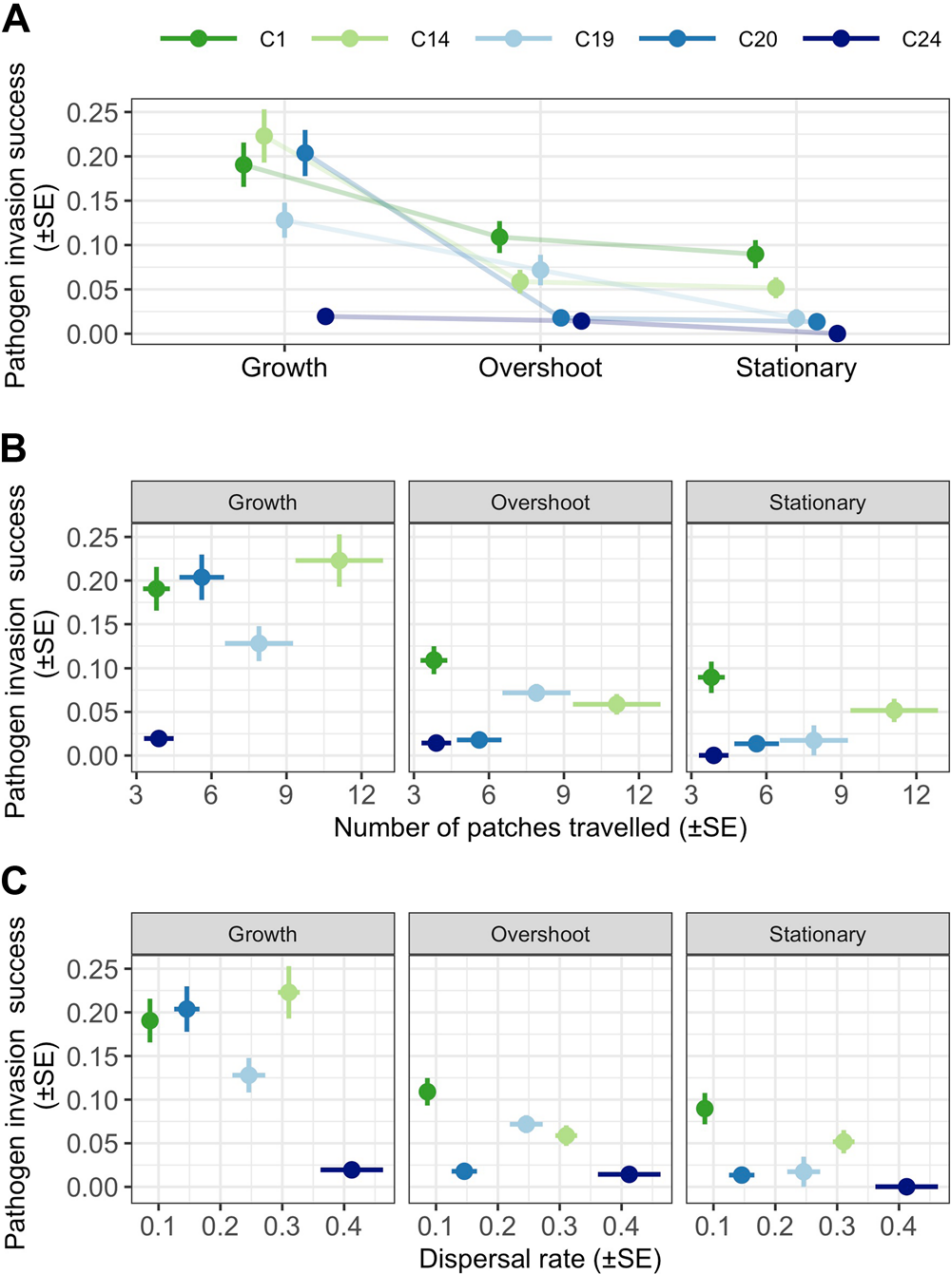
Estimates of integrative pathogen invasion success versus the dispersal capacity of each pathogen genotype. Shown are (A) pathogen invasion success (SE) when invading host populations undergoing rapid growth, overshoot, and stationary phase; (B) the relationship between pathogen invasion success and total distance covered when contrasted with each host colonization phase; and (C) the relationship between pathogen invasion success and dispersal speed of infected hosts, as estimated by the slope of the accumulate number of patches traveled versus time in days.

Integrating within‐population invasion steps (steps 1–3, Fig. 1) into a single metric of invasion success highlights the tension that a pathogen faces between within‐population exploitation and between‐population redistribution. Overall, pathogen invasion success was highest when establishing in populations undergoing rapid growth and reduced as the host population colonization progressed (see Fig. 6A). However, the response of each pathogen genotype to the different host population colonization phases varied. For example, pathogen C14, C20, and C1 invaded recently colonized populations with high, indistinguishable success rates (95% CI), whereas pathogen C1 had much higher invasion success than the other pathogens in host populations at overshoot or carrying capacity, and pathogen C24 had low invasion success in all host colonization phases (Fig. 6A).

Comparing the invasion success of each pathogen genotype with their dispersal rate and total distance covered suggested that complex (rather than simple colonizer‐competitor) relationships exist between within‐population performance and dispersal (Fig. 6B,C). For example, when using total distance as our dispersal metric, the genotype with the highest invasion success in a recently colonized host population also had the highest capacity to disperse (C14), whereas the genotype with the highest invasion success in populations at carrying capacity traveled the least (C1; Fig. 6B). Our results are less clear when contrasting dispersal rate; here, pathogen C24 is the most virulent and least successful invader—but has the highest dispersal rate during the short lifespan of their hosts.

## Discussion

Spatiotemporal variation in host demography is commonly observed in metapopulations and during range expansions (Hanski and Gilpin 1991; Phillips et al. 2010). The resulting distribution of host demographic phases enables pathogens to potentially diversify along a colonizer‐competitor axis (Wei and Krone 2005; de Roode et al. 2008; Magalon et al. 2010), such that pathogens that excel at establishing in newly colonized host populations are likely to be less fit in host populations at overshoot or carrying capacity. If such a diversification and trade‐off exist, it has important implications for emerging theory on the evolution of virulence during invasions (Griette et al. 2015; Osnas et al. 2015; Lion and Gandon 2016), but it has rarely (if ever) been demonstrated empirically (but see Kelehear et al. 2012; Phillips et al. 2012; Mondet et al. 2014). By experimentally introducing pathogens into experimental host populations undergoing different phases of colonization, we show that the conditions required for diversifying selection for a pathogen is indeed embedded within the demography of the host population.

Across each stage of the pathogen invasion process (e.g., Fig. 1), we observed significant variation among genotypes and a change in the rank order for invasion traits at each host colonization phase. All pathogens, for example, were more readily able to establish in recently colonized host populations, but the decline in the probability of establishment as host populations aged was felt more greatly by some genotypes (C1 vs. C20, Fig 3A). Also, pathogen C19 and C20 produced more transmission spores when establishing in host populations at overshoot or carrying capacity, whereas all other pathogens had lower spore production in those populations (Fig. 3B). There were likewise significant differences among genotypes in their capacity to generate new secondary infections under different host densities (Fig. 4), as well as their ability to disperse into new patches (Fig. 5). Taken together, we observe highly complex reaction norms for each pathogen in response to host demography, suggesting that not all pathogens are adopting the same invasion and dispersal strategies.

Our integrated metric of invasion success for each pathogen genotype highlights the evolutionary importance of a host population's colonization history for disease outcomes (Fig. 6A). Here, pathogen invasion success was highest in recently colonized host populations, and declined with the subsequent demographic transitions of the host population. This decline in invasion success, however, was not felt equally among all pathogen genotypes. As host populations transitioned toward the stationary phase, not only did the average invasion success decline, but so too did the differences among genotypes. Together, these observations suggest that newly colonized host populations are the most evolutionarily liable for the pathogen, allowing for higher invasion success and greater adaptive potential (i.e., more genetic variability; Connallon and Hall 2018), in concert with a host population that is free from density‐dependent regulation and is rapidly expanding (e.g., Giometto et al. 2014; Fronhofer et al. 2017).

The higher pathogen invasion success in recently colonized populations contrasts with studies of other *Daphnia* and pathogen species, where the likelihood of an infectious outbreak (as estimated by *R*_0_) was maximized in either intermediate (Civitello et al. 2013) or high‐density populations (Dallas et al. 2018). Dallas et al. (2018), for example, demonstrated that secondary infections and epidemic size increase with host density. This result mirrors theory on the role of patch quality in host metapopulations. Here, high‐quality or resource rich patches both attract and support greater host and pathogen densities, facilitating a constant supply of infected carriers across a landscape (e.g., Leach et al. 2016; Becker et al. 2018). In contrast, Civitello et al. (2013) found that pathogens are more likely to invade populations of intermediate density, as foraging interference limits transmission at higher host densities. Our study (and invasion metric) instead highlights the sensitivity of an invading infected carrier to the demography of the host population encountered and the dependence of any response on the pathogen genotype involved. From a pathogen's perspective, therefore, the quality of a patch is not just shaped by host density. It is also affected by the colonization history of the host population, the changes in demography that ensue, and the pathogen's capacity to invade and exploit these different colonization phases. Thus, the conditions of a host population that maximize the initial pathogen invasion may not necessarily be the same that maximize transmission, or the long‐term maintenance in a host population.

The above results, in isolation, suggest that the optimal investment for a pathogen in traits associated with the invasion process would be those that maximize performance in newly colonized host populations. Within a single location, however, such prime conditions are very transient (i.e., Giometto et al. 2014). To be able to routinely exploit newly colonized host populations, a pathogen inevitably requires a high dispersal capacity (Phillips et al. 2010; Hajek and Tobin 2011, sensu Bonte and Dahirel 2016). Here, not all pathogen genotypes showed this capacity. Pathogen C14 most closely matched our profile of a “fugitive genotype”; it was most successful at invading newly colonized host populations and better able to disperse. In contrast, pathogen C1 maintained its competitive advantage at all stages of host colonization, but was also unlikely to take advantage of any greater success in newly colonized patches due to its low dispersal (Fig. 5). Thus, rather than a simple divergence in pathogen invasion strategies along a single colonizer‐competitor axis, we instead identify a variety of relationships between pathogen invasion success and dispersal, suggesting that the tension between colonization and competition might be optimized in various ways.

Emerging theories on the short‐term evolution of virulence during a spatially spreading epidemic center on whether a trade‐off between virulence and dispersal occurs (see Griette et al. 2015; Osnas et al. 2015). In our study, infection always reduced dispersal distance (Fig. 5), but this reduction was highly variable among genotypes (e.g., C1 vs. C14), and correlated with lifespan of the host. Thus, pathogen genotypes that allow for high host‐facilitated spread are likely to be less virulent, because infection typically acts to reduce host lifespan (Gipson and Hall 2018). Our results support the subset of spatiotemporal models predicting that the need for dispersal will constrain the evolution of virulence (see Osnas et al. 2015 and discussion in Altizer et al. 2011). In our system, then, we would expect evolution to favor less virulent pathogens at the edge of a range expanding host population (Osnas et al. 2015), and in analogous metapopulations.

Our results suggest a number of predictions for infectious disease evolution that are likely testable in the field (see also Hall and Mideo 2018). In nature, *Daphnia* populations experience large fluctuations in population size due to rapid responses to environmental conditions (i.e., winter thaw Caravalho and Crisp 1987), or the colonization of new rock pools founded by individuals in a metapopulation setting (Pajunen and Pajunen 2003; Haag et al. 2006). We suggest that both recently colonized patches and the outer edge of a diffusing population are more likely to be founded by the more dispersive, uninfected *Daphnia*. Our results also suggest that the pathogen genotypes that arrive first in a new population are likely to have relatively low virulence, but eventually, these fugitive genotypes will be out‐competed by the later arriving, but competitively superior pathogen genotypes.

In summary, our work demonstrates the potential for diversifying selection to operate within a single pathogen species across a gradient of host colonization. Although we might expect this diversifying selection to generate a clear spectrum of pathogen genotypes along a colonizer‐competitor gradient, our results indicate that a complex array of pathogen strategies are possible, depending on the demographic conditions of their host population. Given the inevitable variation in host demographic stages in the real world (driven by invasion front or metapopulation dynamics), these selection differences could maintain a diversity of pathogen invasion strategies across space and time. Whether pathogens in these spatiotemporally dynamic situations evolve to become more or less virulent will depend on the trade‐off between pathogen virulence and dispersal, and our work reiterates that this trade‐off may often be a real fundamental constraint on virulence evolution.

Associate Editor: K. Lythgoe

## Supporting information


**Figure S1**. Total host population size over time (days) since first colonizing disperser arrives in a given patch (patch 1, 2, and 3 in different blue nuances).
**Table S2**. ANOVA results predicting the effect of population colonization phase and pathogen genotype on A) pathogen establishment and B) spore load in infected individuals that are establishing in a given host population.
**Table S3**. ANOVA results predicting the probability of infection at different host densities and pathogen spore doses.
**Table S4**. ANOVA results predicting square root transformed total number of patches dispersed.
**Table S5**. ANCOVA results from a linear mixed effect model predicting accumulated number of patches dispersed over time.
