# Supplementary material for: Infection in patchy populations: Contrasting pathogen invasion success and dispersal at varying times since host colonization
Source: Evol Lett. 2019 Sep 24;3(5):555–66. doi: 10.1002/evl3.141 (PMC6791296; doi:10.1002/evl3.141)
Supplement: Supplementary file 1 — Figure S1. Total host population size over time (days) since first colonizing disperser arrives in a given patch (patch 1, 2, and 3 in different blue nuances). Table S2. ANOVA results predicting the effect of population colonization phase and pathogen genotype on A) pathogen establishment and B) spore load in infected individuals that are establishing in a given host population. Table S3. ANOVA results predicting the probability of infection at different host densities and pathogen spore doses. Table S4. ANOVA results predicting square root transformed total number of patches dispersed. Table S5. ANCOVA results from a linear mixed effect model predicting accumulated number of patches dispersed over time. [file EVL3-3-555-s001.pdf]

Supplementary Material

Infection in patchy populations: contrasting pathogen invasion success and dispersal at varying times since host colonisation

Louise S. Nørgaard[1*], Ben L. Phillips[2] & Matthew D. Hall[1]

[1]School of Biological Sciences and Centre for Geometric Biology, Monash University, Melbourne 3800, Australia

[2]School of BioSciences, University of Melbourne, 3010 Parkville, Victoria, Australia

*Corresponding author: louise.noergaard@monash.edu

Section A: Standard experimental conditions

To prepare experimental animals, *Daphnia* females were isolated from stock cultures and reared under standard conditions (60-mL jars filled with 50 mL artificial *Daphnia* medium (ADaM, Klüttgen et al. 1994; modified by Ebert et al. 1998)) for three asexual generations. Animals were fed daily with green algae *Scenedesmus sp.* following standard feeding procedures, gradually increased from 0.5 to 5 million cells, to accommodate the developing animal's needs. All animals were maintained in a controlled temperature room under standard conditions (20°C, 16L:8D light cycle). Individuals were transferred to fresh ADaM twice a week and trays were rearranged daily to minimise any positional effects. Infected animals for use in subsequent invasion experiments were generated by exposing *Daphnia* housed individually in 60-mL jars (filled with 20 ml ADaM) to 20,000 spores at ages 3 and 4 days (i.e., 40,000 spores in total).

Kluttgen, B., Dulmer, U., Engels, M., Ratte, H.., Klüttgen, B., Dülmer, U., et al. (1994). ADaM, an artificial freshwater for the culture of zooplankton. *Water Res*. 28:743–746.

Ebert, D., Zschokke-Rohringer, C.D. & Carius, H.J. (1998). Within–and between–population variation for resistance of *Daphnia magna* to the bacterial endoparasite *Pasteuria ramosa*. *Proc. R. Soc. B.* 265:2127–2134.

Section B: Characterising host colonisation dynamics

To test for the repeatability of the colonisation process in experimental populations, we used a generalised additive model to predict how changes in total population size vary with the time since the first colonising disperser arrives in a given patch (modelled as a thin plate spline), and independently the patch number of an interconnected three-patch system (via the mcgv package (Woods 2006)). We found a significant effect of time since colonisation on population size (e.d.f. = 6.725, F = 29.63, $p < 0.001$) and no difference in average population size with patch (d.f. = 2, F = 1.012, p = 0.370). We then compared this model to one where the change in population size with time was allowed to vary by patch. We found that allowing the trends to vary by patch did not significantly improve the fit of the model (d.f. = 3.703, F = 1.783, p = 0.151) thus confirming the repeatability of colonisation phases across patches (Figure S1).

Three characteristic colonisation phases were evident from the changes in population size since the first colonising disperser arrives, as discussed in the results (rapid growth = 0 to 20 days, overshoot = 20 to 40 days, and carrying capacity = 40 days onwards). To confirm that the population dynamics at each phase were indeed different, we regressed total population size against time for each phase category individually. We found that during the rapid growth phase, populations size increased with time since colonisation (slope = 8.18, SE = 1.623, $p < 0.001$), but were readily reduced (reached a peak and then reduced) during the overshoot phase (negative slope = -3.012, SE = 0.992, p = 0.008), and remained stable during the stationary phase (slope = 0.435, SE = 0.573, p = 0.461). Thus, in our analyses, we chose to partition host colonisation dynamics into three broad phases; rapid growth, overshoot, and carrying capacity.

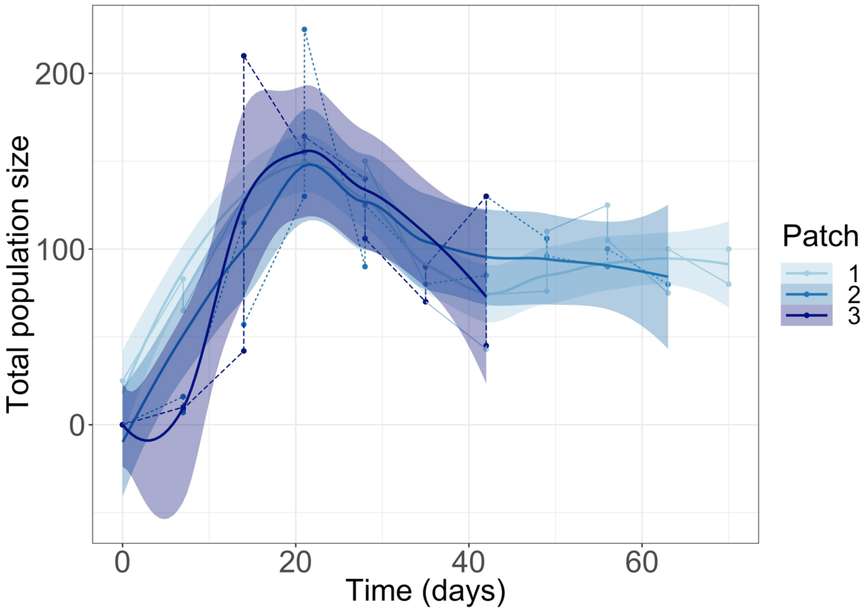

Figure S1 Total host population size over time (days) since first colonising disperser arrives in a given patch (patch 1, 2 and 3 in different blue nuances). Dots represent raw measures and lines represent a fitted generalised additive model with days modelled as a thin plate spline.

Woods, S. N. (2006). *Generalised Additive Models – An Introduction with R*. CRC/Chapman & Hall, New York.

Section C: Pathogen establishment and within-host proliferation

Table S2 ANOVA results. Effect of population colonisation phase and pathogen genotype (Gp) on: A) pathogen establishment; and, B) spore load in infected individuals that are establishing in a given host population. This data, and the measures of proportion of individuals that become infected at a given host density and pathogen spore dose, is used to calculate the transmission coefficient in the integrated metric of pathogen invasion success. (* $p < 0.05$, ** $p < 0.01$, *** $p < 0.001$).

**A) Probability of establishment**	χ^2	**d.f.**	**P-value**	**Sign. code**
Colonisation phase	59.853	2	<0.001	***
Gp	55.889	4	<0.001	***
Colonisation phase x Gp	31.853	8	<0.001	***
**B) Spore load**				
Colonisation phase	16.761	2	<0.001	***
Gp	37.693	4	<0.001	***
Colonisation phase x Gp	20.310	8	<0.01	**

Section D: The potential for secondary infections

Table S3 ANOVA results predicting the probability of infection using a generalised mixed-effect model using spore dose (low, medium and high) and pathogen genotype as fixed effects, population density (individual per litre) as a continuous covariate and experimental jar as a random effect. (* $p < 0.05$, ** $p < 0.01$, *** $p < 0.001$).

**Effect**	**d.f.**	χ^2	**Pr (>χ^2)**	**Sign. code**
Dose	2	1.128	0.569	
Pathogen (Gp)	4	15.959	0.003	**
Density	1	37.121	>0.001	***
Dose x Gp	8	11.238	0.189	
Dose x Density	2	6.522	0.038	*
Gp x Density	4	8.118	0.087	.
Dose x Gp x Density	8	9.216	0.324	

Section E: Experimental measures of pathogen dispersal

Table S4 ANOVA results for the full model predicting square root transformed total number of patches dispersed using infection treatment (pathogen genotypes and uninfected control, Trt) as fixed effect. (* p < 0.05, ** p < 0.01, *** p < 0.001).

**Total patches**	**d.f.**	**F-value**	**Pr (>F)**	**Sign. code**
Infection treatment (Trt)	5	35.662	<0.001	***
Residuals	111			

Table S5 ANCOVA results from a linear mixed effect model with accumulated number of patches as a continuous response variable and time (in days) as a continuous covariate, and infection treatment (pathogen genotypes and uninfected control) as fixed effects and individual id as random effect. (* p < 0.05, ** p < 0.01, *** p < 0.001).

**Dispersal rate**	**d.f.**	χ^2	**P-value**	**Sign. code**
Infection treatment (Trt)	5	35.779	<0.001	***
Day number	1	218.044	<0.001	***
Day number x Trt	5	445.114	<0.001	***

Section F: An index of pathogen invasion success

The aim of this model is to estimate pathogen invasion success at the three identified characteristic host population growth phases: rapid growth, overshoot and stationary phase (Figure 2). By integrating our empirical measures of pathogen life-history traits (sensu Figure 1) into a simple SI framework that is robust to the density of hosts that a pathogen encounters, we can investigate how pathogen invasion success varies depending on the time since colonisation of the host population.

First, we were interested in modelling the probability that an infected individual or infective propagule invades a healthy host population and survives long enough to become infective (produces mature spores) and thus generate new secondary infections. Here, we let E be the probability that an introduced individual survives long enough to become infective, and L the number of infective spores that an infected individual produce, given that it survives long enough for mature spores to be produced. We can then estimate the number of spores likely introduced into the population for each of our five experimentally tested pathogen genotypes, with accompanying levels of uncertainty, calculated by 1000 bootstrap iteration as integrated via the boot function in R:

$$\hat{P}_{dg} = L_{dg}E_{dg} \qquad \textbf{\textit{Equation S.1}}$$

Here, d and g denote host population colonisation phase and pathogen genotype, respectively. P_{dg} can be estimated in our case from the data of establishment and proliferation generated in section 2 of the main methods.

Next, the number of new infections produced by release of a given number of spores in the environment depends on the density of susceptible hosts (S), the number of spores introduced (P), and the transmission coefficient, β. Thus, in any given host population, variation in pathogen infection is driven entirely by variation in β and P.

$$I_{dg} = \beta_g P_{dg} S$$ ***Equation S.2***

From this, and the data from section 3 – on number of new infections (*I*) for each genotype across a range of spore loads (*P*) and host densities (*S*), we can now estimate β_g and accompanying uncertainty levels by bootstrapping the mean:

$$\hat{\beta}_g = \frac{1}{n} \sum_{ijk} \frac{I_{gk}}{P_{ik} S_{jk}}$$ ***Equation S.3***

where i and j represent treatment levels for spore load and host density respectively, k represents replicates within treatment levels, and n is the total number of replicates in the experiment.

Finally, for the purpose of calculating a standardised metric of pathogen invasion success, w_{dg}, we set $S = 1$, thus capturing how different colonisation phases influence the relative capacity of a pathogen genotype to initially invade such a patch, irrespective of the variability in host numbers within each population. Then our pathogen invasion success metric for each pathogen genotype and host population colonisation phase is:

$$w_{dg} = \beta_g P_{dg}$$ ***Equation S.4***

Finally, we contrasted pathogen invasion success at each host population colonisation phase with our experimental measures of host facilitated dispersal, to look for evidence of diversifying selection and trade-off dynamics among the different pathogen genotypes.
